# Kinetic Investigation by Batch Processing of Film Growth during the Electrodeposition of Titanium from Ethaline

**DOI:** 10.1002/open.202500311

**Published:** 2025-06-20

**Authors:** Katarzyna Grubel, Diana B. Horangic, Steven Livers, Christopher J. Chancellor, Riah Burnett, Bailey Byrd, Bethany Lawler, Christina Arendt, Lance R. Hubbard

**Affiliations:** ^1^ National Security Pacific Northwest National Laboratory Richland WA 99354 USA; ^2^ Dept. of Chemistry Alabama State University Montgomery AL 36104 USA; ^3^ Q‐20 Los Alamos National Laboratory Los Alamos NM 88754 USA

**Keywords:** cyclic voltammetry, deep eutectic solvents, electrochemical impedance spectroscopy, electroplating, large dataset processing, titanium

## Abstract

Titanium electrodeposition as a technological advance has been out of reach due to the poor film quality obtained when using previously developed methods. This study shows that the electrodeposition of Ti is possible and feasible from the deep eutectic solvent ethaline. Moreover, this study shows through batch analysis that this deposition has temperature‐dependent aspects that alter the film deposition kinetics upon modest heating. This ultraviolet–visible spectroscopic study is highlighting the more facile reduction of Ti^4+^ to Ti^3+^ and then to Ti^2+^ at elevated temperatures. Understanding the underlying kinetics of electrodeposition opens up new venues for the application of this important metal.

## Graphical abstract

The electrodeposition of titanium in ethaline appears to proceed according to established kinetics in prior literature except above 50 C, where the production of intermediary ions from the polyol process begins to play a large role in the deposition kinetics.

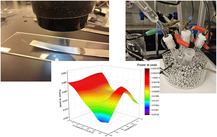

## 1 Introduction

Titanium electrodeposition as a technological advance has been out of reach due to the poor film quality obtained when using previously developed methods.^[^ ^1^, ^2^, ^3^ ^]^ Titanium (Ti) is a refractory metal of crucial importance to the medical, automotive, and aerospace industries, deposition of which on various metallic and nonmetallic substrates leads to their increased strength, inertness, and corrosion resistance.^[^ ^4^, ^5^, ^6^ ^]^ Generally, electroplating has remained out of reach for titanium and similar metals as their reductive bias is greater than the electrolysis bias of water; thus, only nonaqueous solutions could be used.

Deep eutectic solvents (DESs), a category of nonaqueous organic solvents, are being intensely investigated for their applicability, usefulness, and low costs.^[^ ^7^, ^8^ ^]^ DESs have been previously used for electrodeposition of a multitude of metals and alloys,^[^ ^9^ ^]^ but to date, there is no report of successful electrodeposition of refractory metals from them. As late as 2017, it was postulated to be nearly impossible to deposit refractory metals such as Ti from ionic liquids (a similar organic solvent).^[^ ^1^, ^10^ ^]^ In 2021, it was shown that Ti sheet could be electropolished in the DES ethaline.^[^ ^11^ ^]^ However, it was long noted in the literature that while reasonable electrodeposition of titanium can be accomplished in molten salts, electrodeposition at near‐ambient temperatures almost always led to the deposition of titanium oxides.^[^ ^10^, ^12^, ^13^ ^]^ Thus, the electropolishing result was promising, but more work was needed to demonstrate that ethaline can be an effective solvent for titanium electrodeposition.

Previously, particular steps for Ti(IV) reduction have been proposed based on the electrochemical potentials,^[^ ^12^, ^14^, ^15^ ^]^ but they lacked a secondary method that would confirm the specific suggested sequence. As there is a kinetic mechanism discrepancy by method (i.e., molten‐salt reduction, ionic liquid reduction, or computational), a large dataset (300+ individual spectra) centered around the cyclic voltammograms was explored in this work to evaluate thermally driven mechanistic changes. While the mechanism of this deposition can be mostly resolved using reduction potentials, there are no reports supporting an exact layer growth mechanism, that is, whether a monolayer is formed first or the deposition proceeds through a different pathway such as island growth.

The electrochemical impedance spectroscopy (EIS) technique has been used for the characterization of film deposition processes.^[^ ^16^ ^]^ It has also been used to ascertain the growth mechanism upon film deposition as well as the destruction of the electrodes and electrodeposition.^[^ ^17^, ^18^, ^19^ ^]^ It has been shown that EIS is sensitive to the porosity and electrical resistance of the working surface of the electrode.^[^ ^20^ ^]^

In this article, we describe the successful electrodeposition of Ti on a stainless‐steel substrate using the DES ethaline. As both direct reduction and multistep kinetic models have been shown simultaneously for Ti reduction from the 4+ ion state, this work elaborates on a thermal deviation unique to the DES solvent. We characterize the electrodeposition film growth kinetics and use optical spectroscopy to evaluate the in‐situ presence and identity of titanium ions in the solution. Based on our previous experiences, we initially hypothesized that: 1) Electrodeposition of Ti progresses through the formation of “islands” that then grow to form a complete layer, and 2) electroless polyol reduction of Ti(IV) ions increases the presence of Ti(III) and Ti(II) at temperatures greater than 60 °C.

In this study, we have shown that the electrodeposition of Ti is possible and feasible from the DES ethaline. Moreover, we have shown that this deposition has temperature‐dependent aspects that alter the film deposition kinetics upon modest heating. Our ultraviolet–visible (UV–vis) spectroscopic study has highlighted the more facile reduction of Ti^4+^ to Ti^3+^ and then to Ti^2+^ at elevated temperatures.

## 2 Experimental Section

### 2.1 Materials

The chemicals used in the current study are listed in **Table** **1**. All chemicals were used without further purification.

**Table 1:** List of chemicals used in this study.

Name	CAS	Purity [%]	Supplier
Choline chloride	67‐48‐1	98+	ThermoFisher Sci.
Ethylene glycol	107‐21‐1	99.8	Acros Organics
Lithium chloride	7447‐41‐8	99.98	Sigma Aldrich
Titanium tetrachloride	7550‐45‐0	99.9	Acros Organics
Titanium tetrabromide	7789‐68‐6	98	Sigma Aldrich
Titanium tetrafluoride	7783‐63‐3	98	Alfa Aesar
Isopropyl alcohol	67‐63‐0	99.9	Fisher Scientific

### 2.2 Electrochemical Solvent and General Electrical Procedure

Ethaline was synthesized from choline chloride and ethylene glycol (1:2 molar ratio) by combining them in the inert atmosphere of argon and stirring (300 RPM) and then stored under a slow constant sparge of Ar. The purity of the argon sparge (1 L/day) was kept below 4 part‐per‐million atomic for water and oxygen contamination. This value was used to maintain a “dry” DES as the presence of water was observed to directly increase the amount of oxide present in the resultant metal depositions.

The general procedure for electrochemical experiments: In a 20 mL scintillation vial, 20 mL of DES was combined under argon purge gas with lithium chloride (10 mg) and a source of titanium (0.334 g TiF_4_, 0.3 mL TiCl_4_, 0.613 g TiBr_4_). Solids were added directly into the vial, while TiCl_4_ was drawn through a 5 μm polyvinyl difluoride membrane syringe filter into a disposable syringe and then discharged into DES solution. Obtained suspensions were put under a blanket of argon and sonicated at 60 °C, resulting in a clear homogeneous solution. The bath compositions were:

DES, 4‐732 mL depending on the plating cell's requirements

11.8 mM LiCl (initially determined necessary from literature, the concentration was experimentally optimized)

A single titanium 4+ ion source: 1) 133 mM TiF_4_ (slightly oversaturated; crystals were allowed to accumulate in the bottom of the plating cell); 2) 137 mM TiCl_4_ (saturated; added slowly over 1 min as there was significant heating upon addition to DES); and 3) 83.5 mM TiBr_4_ (saturated; the bromide ion was not dissolved well in DES even under significant sonication on the hour time scale).

Next, the solution was transferred to a 5‐port Gamry Inc. small Dr. Bob's Cell^TM^ (cleaned with Alconox, a base cleaning agent) and vendor‐supplied fittings. The cell was inserted into a glass bowl with metallic thermal‐transfer beads and placed on a hot plate where the temperature was set from 30 to 90 °C during separate runs. Electrochemistry experiments were set up in the cell equipped with a vendor‐supplied aqueous silver/silver chloride reference electrode, separated from the reaction chamber by a DES‐filled bridge tube and vendor‐supplied glass frits, graphite counter electrode, and stainless‐steel (316, McMaster Carr product: 2317K275) working electrode. The working electrode was polished with 400 A sandpaper and wiped with a Kimwipe^TM^ immediately before its insertion into the corrosion cell. During electroplating, the reaction was kept under a constant blanket of Ar.

The electrochemical measurements were performed with a Gamry Inc. Interface 1010 E potentiostat. Plating occurred by square wave pulse plating of 0.1 s at −1.94 V and 0.05 s at −0.194 versus Ag/AgCl reference (i.e., the rest “0” potential when measured against a standard hydrogen reference electrode). For data plotted against the reference voltage, the experimentally determined conversion value of 0.194 V was used to adjust the bias from the aqueous silver/silver chloride electrode to a standard hydrogen electrode (SHE). The conversion to SHE was performed to enable a more ready comparison between plating methods in the prior literature and to standardize the bias between electrochemical analytical methodologies. After the electrodeposition experiment was completed, the cathode was removed and rinsed with isopropanol and then submerged and sonicated (40 W, 40 kHz) in isopropanol for 5 min. After sonication, the cathode was dried under a light argon stream and placed into a vacuum desiccator until imaging and/or electrical resistance measurements were taken.

### 2.3 Optical Experiments Procedure

Optical experiments were performed in the argon‐filled glove bag in a 20 mL scintillation vial equipped with a custom, 3D‐printed cap (Figure S1, Supporting Information). Background absorption of DES was recorded before each measurement. General procedure: 10 mg of LiCl was weighed into a 20 mL scintillation vial. Next, DES was added, and the vial was blanketed with argon. TiCl_4_ was drawn through the filter and discharged into the DES solution. The obtained suspension was put under a blanket of argon and sonicated (40 kHz, 40 W) at 60 °C, resulting in a clear, homogeneous solution, which was taken directly into the glove bag. Samples were heated to desired temperatures using a hot plate, and the progress of reactions was monitored using an optical exposure chamber. Data were collected using Ocean Optics OceanView software at regular intervals (1 or 60 s).

### 2.4 Measurement and Characterization

Electrochemical measurements were carried out using Gamry E1010B Potentiostat. The specific settings for each measurement were recorded in the data headers; example data headers for each electrochemical measurement type are provided in the supplemental information, and electrochemical stability measurements are presented in Figure S2, Supporting Information. Electrical resistance evaluation of the plated films and reference standards was accomplished with a Keysight B258A electrometer and vendor‐supplied tri‐ax cabling and alligator clips.

Scanning electron microscopy (SEM) was performed using a JEOL IT‐800 Field Emission Scanning Electron Microscope, coupled with an Oxford 80 X‐Max energy dispersive spectrometer (EDS). Variable accelerating voltages ranging from 5 to 30 kV were used. Scanning transmission electron microscopy was done on a JEOL JEM‐ARM20°CF ACCELARM spherical aberration corrected (Cs) microscope operated at 200 kV using a JEOL Centurio SSD‐EDS detector with a solid collection angle of 0.9 sr.

X‐ray fluorescence was measured with a Bruker Scientific S2 PICOFOX total reflection X‐ray fluorescence (TXRF) spectrometer. Custom acrylonitrile butadiene styrene‐printed dishes were used to hold the coupons in the TXRF. All samples were measured for 1 hr with a molybdenum cathode and a nominal current of 60 microamps. All data were normalized to the intensity of the iron response to allow for comparison between samples.

Optical UV–vis absorbance data were recorded with an Ocean Optics OCEAN HDX spectrometer with vendor‐supplied fused silica optics and cables. All Ocean Insights products were selected for maximum light collection from 195 to 850 nm. The optical cell was purchased from Thor Labs Inc. (model CVH100) and connected via SubMiniature A (SMA) collimating lenses with fused silica optics (models CVH100‐COL, LA4647).

### 2.5 Data Analysis

Optical/SEM images were analyzed with Image J v1.53. Electrochemical data (excluding cyclic voltammetry [CV]) were analyzed with Gamry's EChem Analyst software.

Collected CV data were analyzed using a custom‐developed algorithm for batch processing of electrochemical data.^[^ ^21^ ^]^ The analysis methodology is provided in the supplemental information. X‐ray fluorescence (XRF) data were analyzed using Bruker's PICOFOX control/analysis software. Optical emission data were analyzed with Microsoft Excel after being recorded by Ocean Optics’ OceanView control software.

The data processing of the 600+ CV scans was performed according to the methodology detailed in the supplemental information. In short, each CV scan was background subtracted over the 2 areas of interest, and the response parameters, such as beginning points, end points, inflection points, center of the response, and integrated response area, were correlated to the deposition conditions to evaluate the influence of the process parameters on the mechanistic responses present in the CV.

## 3 Results

### 3.1 Initial Evaluation

**Figure 1:**
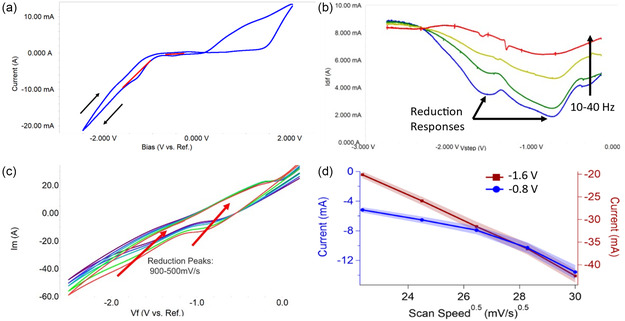
a) Example cyclic voltammogram with the regions of titanium reduction denoted by red lines. b) square wave voltammograms taken with scan speeds from 10 to 40 Hz. Responses from the two observed reductive reactions are demarked. c) Scan speed comparison of cyclic voltammograms from 500 to 900 mV s^−1^. d) A plot of the centers of the reduction responses in Figure 1c showing a linear trend from the response occurring at about −1.64 V (red) and a nonlinear influence in the response occurring at −0.8 V (blue).

During the electrodeposition of titanium on steel, two reduction responses were seen, as evidenced by CV (**Figure** **1**a). The deposition was then analyzed by square wave voltammetry to confirm the reduction biases with minimized capacitance. The reductions appear to be centered around −0.8 and −1.6 V (adjusted to be vs. a SHE), as shown in Figure 1b. The initial reduction response (−0.8 V) occurred over a very broad bias range, warranting further investigation. CV of both responses was conducted from 10 to 10 k mV s^−1^ scan speeds, with the cell providing ion transfer data from 10 to 1 k mV s^−1^. The higher scan speeds had nonlinearities, as shown in Figure 1c. The center of the reduction biases was plotted against the root of the scan speed to evaluate the diffusive nature of the reduction. As shown in Figure 1d, the reduction to metal occurring at −1.6 V is linear, while the intermediary reduction response (−0.8 V) has nonlinear aspects at higher scan speeds. Obtained samples were then imaged and chemically analyzed to verify that deposition was taking place.

### 3.2 Imaging and Chemical Analysis

**Figure 2:**
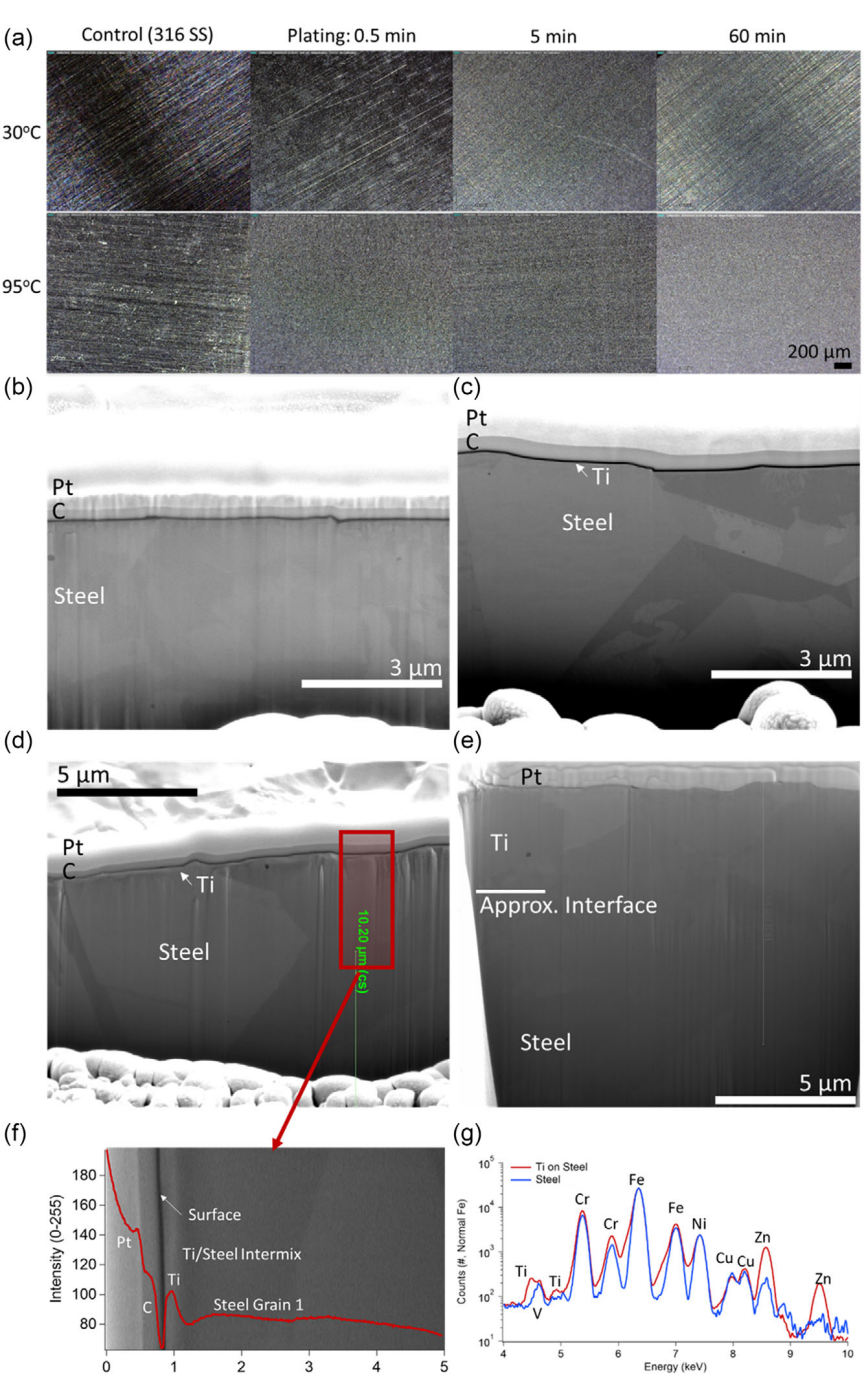
a) Optical image set of the film evolution with temperature and deposition time. b) 20kX SEM cross‐section of the steel substrate. c) 20 kX SEM cross‐section of the titanium deposition after 30 s of deposition and at 30 °C. d) 35kX SEM cross‐section of the titanium deposition after 5 min of deposition and at 90 °C. e) 35 kX SEM cross‐section of the titanium deposition after 1 hr of deposition and at 90 °C. f) Intensity analysis of the 5 min plated cross‐section (with the area marked in Figure 2d), with a 50kX SEM background inset for layer perspective. Each layer is defined by image intensity. g) XRF analysis of the 1 hr plated sample versus a steel control.

Optically, at near‐ambient temperatures, the evolution of the titanium deposition appears to happen by island growth on the seconds time scale, with full film evolution proceeding after that, as shown in **Figure** **2**a, top panel (30 °C). At higher temperatures, the island growth mechanism appears to be supplanted by a conformal layer‐by‐layer deposition, as shown in Figure 2a, lower panel (95 °C). To evaluate this, the films were cross‐sectioned for scanning electron analysis.

Figure 2b shows steel with caps of carbon and platinum used to illuminate the surface. After 30 s of electroplating, the surface appears rougher, which may indicate the presence of islands of titanium (see Figure 2c). After 5 min of plating, a several hundred nanometer‐thick conformal film is developing on the surface, which is shown in Figure 2d. The intensity analysis of the cross‐section allowed for the conformation that the titanium is forming on the surface, shown in Figure 2f. After 1 h of deposition, the film is about 3–3.5 microns thick with small grains evident at the boundary between the steel and titanium (see Figure 2e).

Last, XRF was used to determine the composition of the film, with titanium appearing to be the only addition when compared to a control signal (see Figure 2g). It should be noted that deviances appear in the XRF signal presented in Figure 2g with regard to chrome, copper, and zinc. These variances were not predictable and were within the vendor‐supplied compositional range for the steel substrates. The only metal appearing upon plating was titanium. The micron‐scale thick films shown in Figure 2e,g were also tested for electrical resistance against titanium and steel reference sheets of similar roughness. The electrodeposited films had resistances in line with those of the titanium sheet standard (about 50% less resistance than the steel substrate used for plating).

To confirm the morphology of the titanium coating and how it forms, secondary ion mass spectroscopy was performed at an outside lab on samples that were plated for short times such as 30 s, and the details are presented in Figure S3, Supporting Information. The plated sample showed the addition of 20–30 micron‐wide islands of titanium that are 50–80 nm thick. It should also be noted that the plating appears to be on top of a 3–5 nm thick chromium‐containing layer, as would be expected for the surface of stainless steel. With these results, EIS was carried out to determine the switch in the plating mechanism from island formation to that of a more layer‐by‐layer growth.

### 3.3 Electrochemical Impedance Spectroscopy

**Figure 3:**
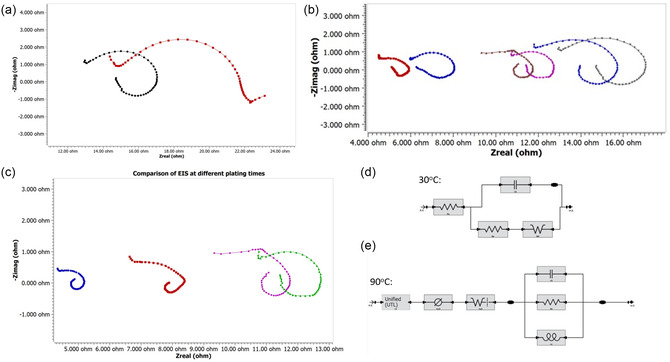
a) Comparison of the initial EIS between 30 °C (red) and 90 °C (black) for electroplating solutions of TiBr_4_ of the same concentration. b) Comparison of Ohmic resistance of Ti coat electrodeposited for 30 sec from different plating baths at 90 °C: TiF_4_ salt (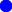
start, 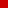
stop), TiCl_4_ salt (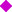
start, 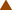
stop), TiBr_4_ salt (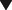
start, 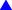
stop). c) Comparison of Ohmic resistance of Ti coat electrodeposited for different periods from the TiCl_4_ salt at 80 °C (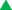 0 sec., 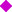 30 sec., 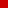 5 min., 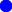 1 h). d) example of simple EIS fitting circuit diagram. e) EIS circuit diagram used to fit EIS post‐Ti layer formation.

To explore the different film depositions seen in Figure 2a, potentiostatic EIS was carried out at two temperatures, three bath compositions, and three different time lengths. Each of the experiments began with a recording of the EIS of the steel electrode before applying current. At low temperatures, the EIS of the SS substrate exhibits an open‐loop feature that allows for distinguishing the kinetic control region and mass transfer‐controlled region (see **Figure** **3**a). However, at an elevated temperature of 90 °C, even before the current is passed through the sample, the appearance of the inductive loop can be seen, indicating that electrode surface modification starts to take place (see Figure 3a).

To better understand the reason for the emergence of the inductive loop (and subsequent alteration in film deposition kinetics), we investigated the influence of different anions on the mechanism of Ti electrodeposition (Figure 3b). In each case, the inductive mechanism was shown to be temperature‐dependent and not ion‐dependent. In each case, initial EIS recorded at 30 °C showed a typical open curve with a clearly distinguishable Warburg region (circuit diagram shown in Figure 3d), but at 90 °C, even initial EIS already shows inductive behavior, as evidenced in Figure 3c with the corresponding circuit diagram shown in Figure 3e. Further attempts at understanding the nonlinearity in the reduction currents seen in Figure 1d were then undertaken.

### 3.4 Cyclic Voltammetry

**Figure 4:**
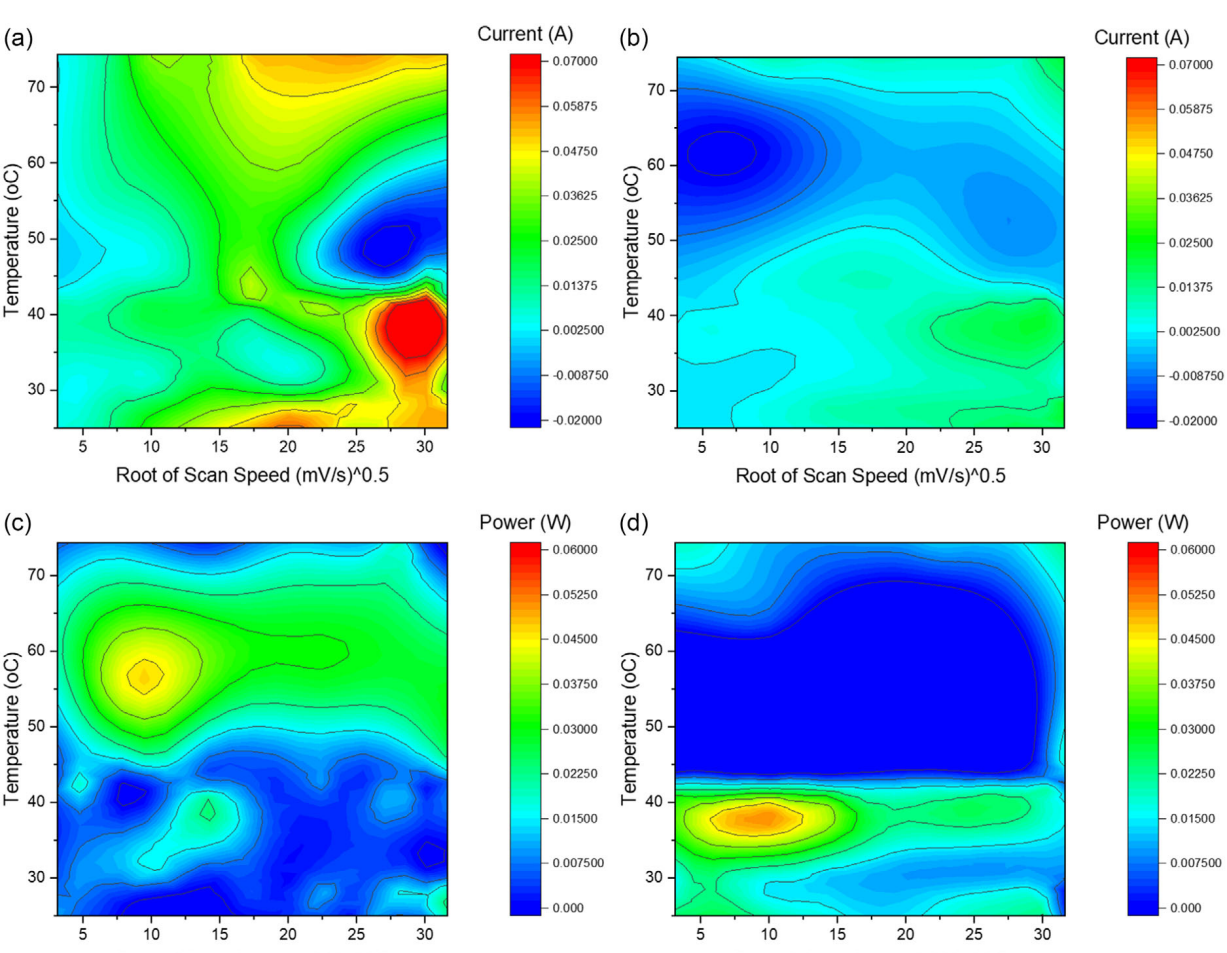
Batch investigation of the CV of the reduction to titanium (IV) chloride. a) A color map denoting the magnitude of the reductive current for the response occurring at about −0.8 V versus a SHE taken at various temperatures and scan speeds. b) A color map denoting the magnitude of the reductive current for the response occurring at about −1.6 V versus SHE taken at various temperatures and scan speeds. c) A color map denoting the magnitude of the integrated charge transfer for the response occurring at about −0.8 V versus SHE taken at various temperatures and scan speeds. d) A color map denoting the magnitude of the integrated charge transfer for the response occurring at about −1.6 V versus SHE taken at various temperatures and scan speeds.

To better understand the reduction kinetics, a dataset of over 1000 CV scans was taken at varying temperatures (25–90 °C) and scan speeds (10–1000 mV s^−1^) for the bath with saturated titanium (IV) chloride. The responses were analyzed for the magnitude of the charge transferred during reduction as well as the maximum magnitude of the reductive current, as shown in **Figure** **4**.

The reductive current change of the initial reductive response is linear for slower scan speeds (5–15 mV^0.5^ s^0.5–1^), as seen in Figure 4a. Also in Figure 4a, higher scan speeds cause nonlinearities to occur (diffusion is no longer the limiting kinetic step). A similar trend is seen in the reductive current of the −1.6 V response seen in Figure 4b. This is true except for one location with higher scan speeds and temperatures from 50 to 60 °C, where both reductive currents are lower than would be expected (see Figure 4a,b).

The total dissipated power (integrated response minus the background signal) of both reductive biases helps explain the trend seen in Figure 4a,b. The power dissipated by the initial reduction at −0.8 V is relatively steady with a maximum response of around 60 °C (see Figure 4c). Lower temperatures produce less of a response than higher ones, as would be expected, until an optimal plating temperature is reached.

The reduction of Ti^2+^ to metal at −1.6 V shows a different trend, with its optimal temperature occurring around 40 °C and an unexpected dearth of signal at higher temperatures, as seen by the large blue region of Figure 4d. This trend is unexpected, as it was hypothesized that the metal reduction would follow a similar trend of an optimal plating temperature being around 60 °C, with slower pulses producing the optimal reductive environment. Since the solvent is relatively transparent across the near‐ultraviolet to near‐infrared wavelengths and titanium ions have different absorbances in this range, in‐situ optical absorption spectroscopy combined with the electrochemical process was undertaken to better understand the unexpected CV results of the metal reduction.

### 3.5 Optical Results

**Figure 5:**
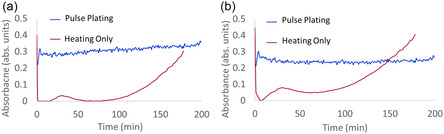
a) A plot of the optical absorbance of 410 nm light, related to the concentration of Ti^2+^ with and without electroplating at 80 °C. b) A plot of the optical absorbance of 520 nm light, related to the concentration of Ti^3+^, with and without electroplating at 80 °C.

To better understand the impact of solvents on the presence and stabilization of titanium ions in the bath, the optical response of each ion was measured with and without the electrochemical pulse plating and at 60 °C, as seen in **Figure** **5**a,b. It should be noted that in the ultraviolet to near‐infrared range, only Ti^3+^ and Ti^2+^ have unique absorbances that cannot be attributed to other ions.^[^ ^22^, ^23^, ^24^ ^]^ Generally, the presence of both intermediary ions is higher during the hot electroplating case as compared to the thermal process only. It should be noted that the Ti^2+^ ion is ever‐increasing in concentration during the electroplating case, suggesting that there may be other production means for this intermediary ion.

In Figure 5a,b for the thermal‐only cases, the presence of both intermediary ions increases in the solution with time at temperature, with Ti^3+^ being more prevalent overall at all times. It is expected, as it requires the addition of just 1 electron to Ti^3+^ versus 2 electrons for reduction to Ti^2+^. For the pulse plating at temperature, the presence of Ti^2+^ is marginally higher and increasing on the minute time scales, while the presence of Ti^3+^ remains constant.

## 4 Discussion

At temperatures above 60 °C, the EIS shows only layer‐by‐layer growth through a diffusive plane (Figure 3b,c,e). rather than one reliant on island growth (Figure 3a,d). This switch is partially explained by the presence of nonlinear influences in the CV of the reductive response centered at about −0.8 V, as shown in Figure 4a. Additionally, based on the EIS result, the initial presence of an inductive loop is evidence of the change in film growth kinetics. It has been shown that for a simple solid electrode (i.e., the metal surface before/during island formation), the impedance of the system should be able to be represented by a simple equivalent circuit with a resistor, capacitor, and Warburg impedance element (accounting for the diffusion‐controlled process), as shown in Figure 3d.^[^ ^25^, ^26^ ^]^ This solid electrode kinetic model fits the deposition kinetics of ambient temperature electroplating. However, while using our setup and heating it above 60 °C, we have found that we had to account for the inductive loop as well as the presence of the porous electrodes. The modeling of the inductive loop and porous electrodes in the diagram in Figure 3e is needed as the deposition kinetics change to a layer‐by‐layer model with the reduction of some ions occurring in narrow pores as the films complete deposition. The use of the united transmission lines accounts for the rate of kinetic reaction in narrow pores of the electrode. The evident change in deposition kinetics is reflected in the optical and electrochemical behavior of the cell.

At temperatures above 60 °C, there is an increase in the current which does not align with a linear trend that would be expected from a diffusion‐limited process, such as is seen by the mostly linear evolution of the metal reduction response in Figure 1c,d, and 4b. Below 60 °C the mechanism corresponds to a similar multipath one as in molten‐salt electroplating,^[^ ^14^ ^]^ and with increasing temperature, the thermally driven reduction causes deviations as more and more intermediary ions are produced by the polyol method. The thermally dependent nonlinearities of the initial reduction response's CV suggest that it is influenced by the production of ions that are not being driven by electroreduction.

UV–vis assessment during the heating of the solution (Figure 5) shows that if no pulse plating is applied, the presence of both intermediate ions (Ti^3+^ and Ti^2+^) increases with time. This is suspected to be due to the polyol reduction,^[^ ^27^ ^]^ as the main component of the solvent is ethylene glycol. This assessment is corroborated by the batch analysis of the CV, which shows nonlinear aspects in current for both reduction peaks, with a particular increase evident for the primary reduction bias (−0.8 V, Figure 4a,b). It should be noted that the presence of electroplating sequence increases the concentration of both intermediate ions; thus, part of their kinetics is driven by electroreduction. Last, an important aspect of the optical signature of the intermediate ions is that while the presence of Ti^3+^ remains relatively stable on the minutes time scale (Figure 5b), the presence of Ti^2+^ increases slightly during pulse plating (Figure 5a). Thus, with the presence of thermal and electrical reduction, there is an overabundance of Ti^2+^ in the bath and available for metal film formation.

The increasing concentration of Ti^2+^ in the bath originating from both electroreduction and solvent‐based polyol reduction appears to be the mechanism by which the film growth kinetics change upon heating, as seen in Figure 2a and 3a,c. In ambient electroplating approaches, there would be a dearth of available ions for metal reduction, meaning that the electrochemical cell must provide all the power (as seen by the increased dissipated power in the lower left‐hand corner of the heat map in Figure 4d). Film growth would then naturally proceed from ideal plating locations (islands).

Taken together, our observations corroborate the hypothesis that the polyol reduction mechanism is influencing the electrodeposition kinetics at temperatures above 60 °C. With an increase in temperature, the concentration of the available Ti^2+^ eventually becomes more than what can diffuse to the surface (i.e., the concentration increases in the bath), thus plating occurs in a more layer‐by‐layer fashion, as is seen in the film depositions at higher temperatures in Figure 2a,c–f and by EIS at higher temperatures in Figure 3b,c,e). Thus, the polyol‐induced production of intermediate ions produces a change in the film growth kinetics that is thermally dependent (as the electroless polyol reduction kinetics are). What this means from the perspective of prior literature is that the multikinetic route has an increased percentage of Ti^4+^ to Ti^3+^ and Ti^3+^ to Ti^2+^ production.^[^ ^14^ ^]^ What is not seen is an increase in metal reduction, which optically would be represented as a diminishing concentration evolution with time of the Ti^2+^ absorbance. It appears that the polyol mechanism can enhance the production of intermediate ions but cannot reduce them to elemental metal, which remains a function of the electrochemical process.

## 5 Conclusions

The electroreduction of titanium has been challenging to implement in the past. In this publication, we have shown that the electrodeposition of Ti on stainless‐steel substrates is feasible from an inexpensive DES ethaline. The reduction kinetics are in line with prior literature at ambient temperatures. Upon modest heating, the influence of the electroless polyol reduction mechanism increases the concentration of intermediate ions, altering the film growth kinetics to a more layer‐by‐layer method. We have highlighted the importance of temperature control on the reaction and have proposed an electrodeposition mechanism that is supported by multiple analytical methods, such as EIS, UV–vis, and SEM. Our work, therefore, may open a pathway to simpler and more environmentally friendly formation of Ti deposits and alloys for use in critical industries and consumer goods. A methodology to electrodeposit active metals such as titanium can revolutionize medical implants, aerospace, and corrosion barriers, providing a way to combine the mechanical properties of the underlying metal with the anticorrosive properties of the active metals.

## Conflict of Interest

The authors declare no conflict of interest.

## Author Contributions

**Katarzyna Grubel**: data curation (equal); investigation (lead); methodology (equal); writing—original draft (equal). **Diana B. Horangic**: investigation (equal); visualization (supporting); writing—original draft (supporting). **Steven Livers**: investigation (equal); writing—original draft (equal). **Christopher J. Chancellor**: investigation (equal); writing—original draft (equal). **Riah Burnett**: investigation (supporting); writing—original draft (supporting). **Bailey Byrd**: investigation (supporting); writing—original draft (supporting). **Bethany Lawler**: methodology (supporting). **Christina Arendt**: conceptualization (supporting); funding acquisition (supporting); methodology (equal); supervision (supporting); writing—review & editing (supporting). **Lance Rex Hubbard**: conceptualization (lead); data curation (equal); funding acquisition (lead); supervision (lead); writing—original draft (supporting); writing—review & editing (lead).

## Supporting information

Supplementary Material

## Data Availability

The data that support the findings of this study are available from the corresponding author upon reasonable request.
