# Supplementary material for: Kinetic Investigation by Batch Processing of Film Growth during the Electrodeposition of Titanium from Ethaline
Source: ChemistryOpen. 2025 Jun 20;14(11):e202500311. doi: 10.1002/open.202500311 (PMC12598788; doi:10.1002/open.202500311)
Supplement: Supplementary file 1 — Supplementary Material [file OPEN-14-e202500311-s001.pdf]

Kinetic Investigation by Batch Processing of Film Growth During the Electrodeposition of Titanium from Ethaline

Katarzyna Grubel[1], Diana B. Horangic[1], Steven Livers[1], Christopher J. Chancellor[1], Riah Burnett[2], Bailey Byrd[2], Bethany Lawler[1], Christina Arendt[3], Lance R. Hubbard[1,3]*

1: Pacific Northwest National Laboratory
2: Alabama State University
3: Los Alamos National Laboratory
*: Corresponding Author, lhubbar1@lanl.gov

Keyword
Deep Eutectic Solvent, Electroplating, Titanium, Electrochemical Impedance Spectroscopy, Cyclic Voltammetry, Large Dataset Processing

Abstract
Titanium electrodeposition as a technological advance has been out of reach due to the poor film quality obtained when using previously developed methods. We have shown that the electrodeposition of Ti is possible and feasible from the deep eutectic solvent (DES) ethaline. Moreover, we have shown through batch analysis that this deposition has temperature-dependent aspects that alter the film deposition kinetics upon modest heating. Our ultraviolet-visible (UV-vis) spectroscopic study has highlighted the more facile reduction of Ti^{4+} to Ti^{3+} and then to Ti^{2+} at elevated temperatures. Understanding the underlying kinetics of electrodeposition will open up new venues for the application of this important metal.

Supplemental Information

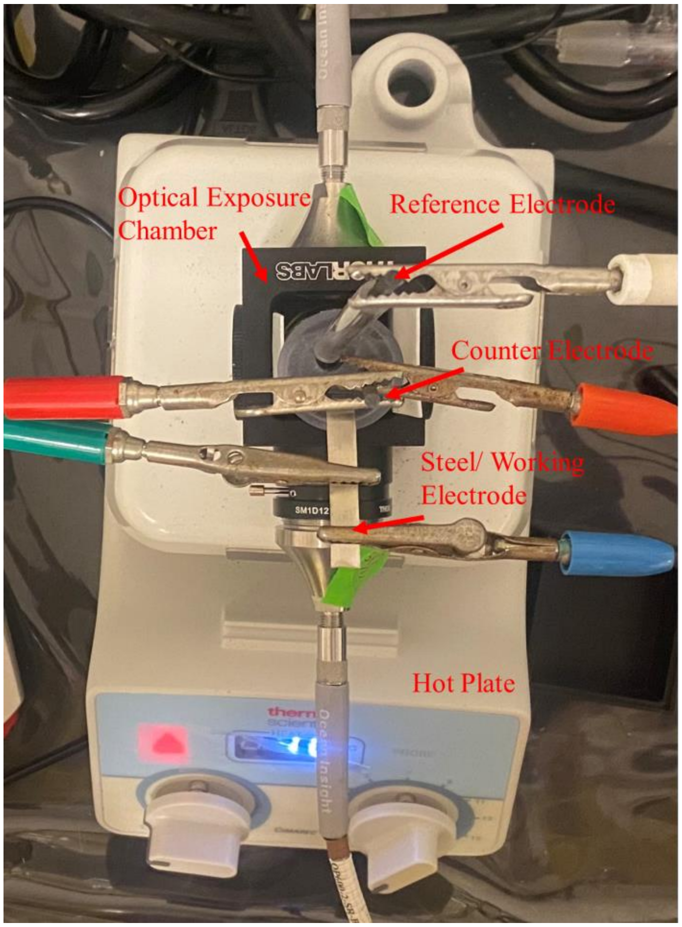

Figure S1. Diagram of the optical and electronic cell used for in-situ optical measurements.

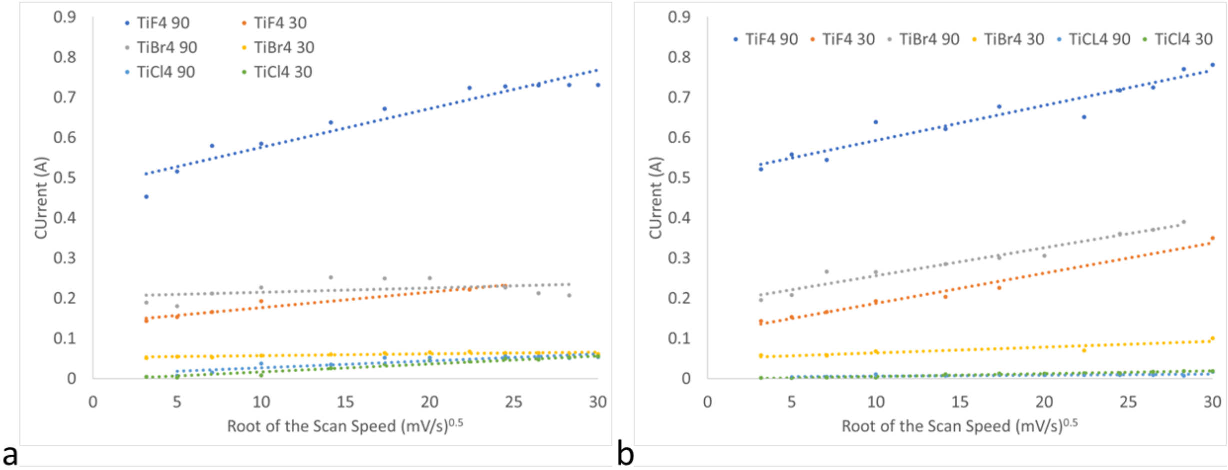

Figure S2. a) Current plotted vs. root of the scan speed for 3 ions of titanium and at 2 temperatures for the reduction current at -0.8V vs. SHE. b) Current plotted vs. the root of the scan speed for 3 counter ions of titanium (IV) and at 2 temperatures for the reduction current at -1.6V vs. SHE.

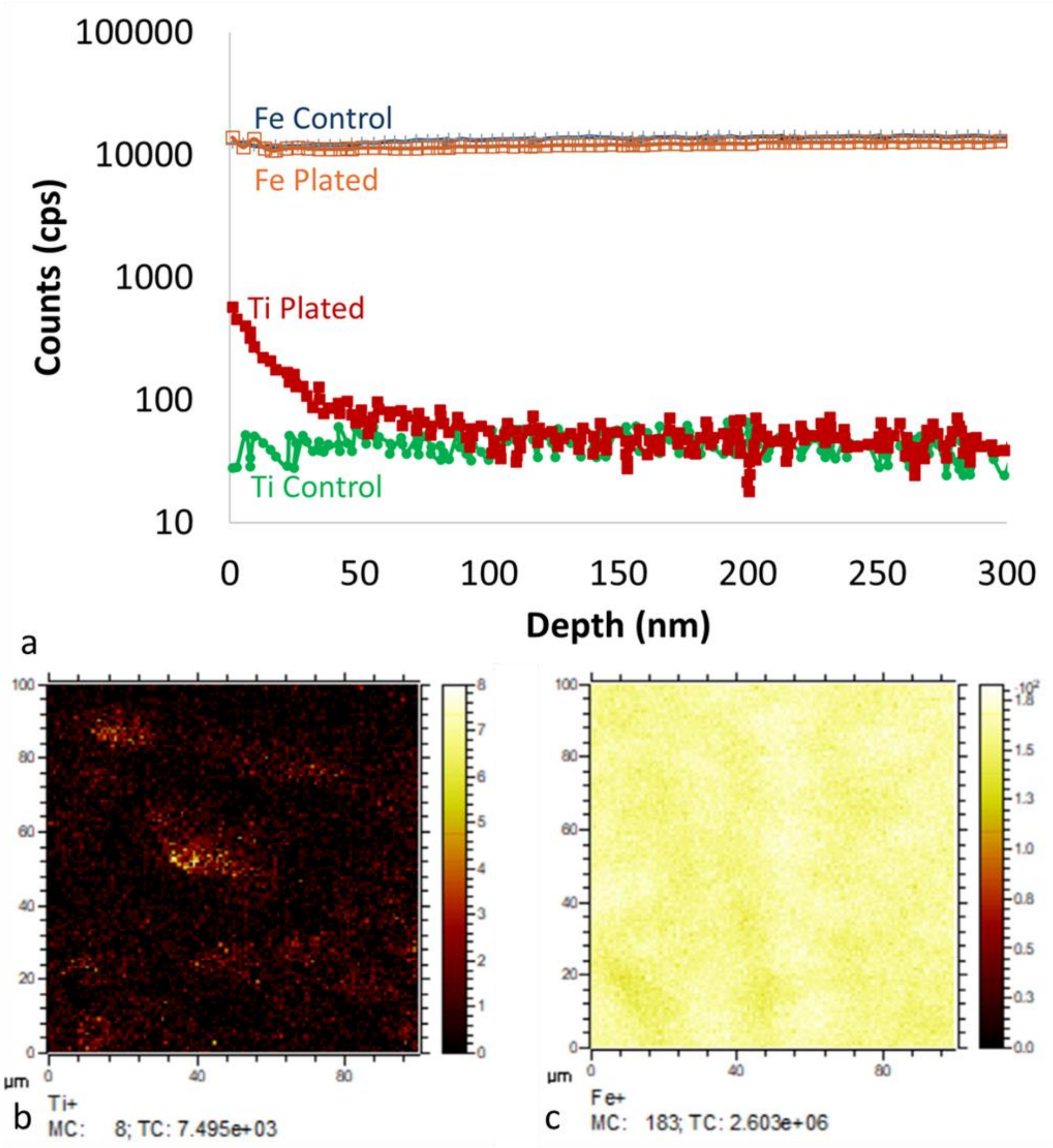

Figure S3. a) SIMS depth profile for a steel control and a short time (30s) titanium plated sample (60^oC) . b) Surface map of the plated sample in (a) showing the evolution of ~20-30 micron wide Ti islands for short plating times. c) the corresponding iron surface map to (b).

CV Data Analysis

Data Organization

Data is organized by 1) different operators, and 2) date of data acquisition. The program reads each operator folder and each date folder, then reads a separate text file for each scan speed (e.g. 10 mV/sec, 50 mV/sec, *etc.*). If the file does not exist in that directory, it is skipped. There is a global dictionary containing the concentration, ion, temperature, and duration of each run that the user defines.

Data Reading and Curve Separation

Text files are read line by line. There is a title in the text file that signals the beginning of all data being taken. After getting the first line of real data, the program cycles through every subsequent line searching for a line that starts with either "Pt" or “CURVE”. This indicates a new run of data and in this line is also either a 2, 3, or 4, which gives the number of the run. The indices dividing each curve are stored. All values of current, voltage, temperature, and time are stored subsequently in a list of rows. This list is converted into a Pandas.DataFrame and stored. Then, the rows of voltage, current, and time are converted to NumPy arrays. A list containing each concatenated NumPy array for each curve is made using the stored indices to divide the one long array into each curve. This is done for voltage, current, and time.

Data Preprocessing

Analysis of trials of scan speed at or above 2000 mV/sec did not yield stable results, as the response of the system (and therefore the analysis) was erratic (i.e., ~1000 mV/s is the max diffusion point of the system). Voltage and current data are shifted for analysis such that both are positive for every point. The order of the array naturally runs “backward” from a higher voltage to a lower voltage; this is reversed by adding the absolute value of the minimum value of each array to every point in the array. These minima are stored and used to convert voltage and current points back to their original value later. The runs are trimmed to run from the minimum to the maximum voltage of the run.

Second Derivative and Maxima, Minima, and Inflection Points Calculation

There is a loop that goes over each curve. The first curve is cut out and never analyzed as it was performed to stabilize the signals from the electrochemical cell. In the loop, voltage and current arrays are stored as X and Y respectively. In preparation for numerical differentiation, the variable dX is calculated where $dX = X[1] - X[0]$. This is the initial “step” of the voltage array used to initialize the process. Multiple derivatives of the data are used to find the start and end of each reduction dip. The start and end of each dip are stored in an array for processing.

Linear Fitting

The pairs of reduction dip start and end indices (i_{start}, i_{end}) are fed to a linear fitting function. These indices are used to generate two pairs of voltage and current. These are the pairs used to generate a line. The slope and intercept are returned by this function and stored for every reduction dip pair.

Integration of Reduction Dips

Integration of each reduction dip is performed using the index pairs, the slope, and the intercept generated before the reduction dip. A numeric integration of the real data is performed using SciPy trapz function. Before the current is integrated, a Gaussian filter with a standard deviation of 4.0 is applied to it. The integration of the line generated by the start and end voltage of the reduction dip is performed analytically. The integral of the line is subtracted from the integral of the original data and stored as the reduction

dip area. The start, end, and halfway voltages of each reduction peak are also stored in their original value. They are converted back using the stored minima mentioned in the Data Preprocessing section.

Data Storage

There are two methods of data storage. The first is to store everything in a long CSV file with a row marking the scan speed. The software generates a separate CSV for each operator. The second method creates an Excel file for each operator. This file has a sheet for every scan speed the operator collected data for, and all dates and the temperature of the run are included in each sheet. It is a condensed Excel file containing the averages of trials performed under the same experimental conditions. It can also include the standard deviation and standard error if specified by the user.

Gamry Cyclic Voltammetry Header Information

EXPLAIN
TAG CV
TITLE LABEL 500Cyclic Voltammetry Test Identifier
DATE LABEL 3/29/2024 Date
TIME LABEL 11:54:52 Time
NOTES NOTES 1 Notes...

PSTAT PSTAT IFC1010-20235 Potentiostat
VINIT POTEN -1.94000E-001 F Initial E (V)
VLIMIT1 POTEN -2.50000E+000 F Scan Limit 1 (V)
VLIMIT2 POTEN 2.00000E+000 F Scan Limit 2 (V)
VFINAL POTEN -1.94000E-001 F Final E (V)
SCANRATE QUANT 4.99999E+002 Scan Rate (mV/s)
STEPSIZE QUANT 1.00000E+000 Step Size (mV)
CYCLES IQUANT 3 Cycles (#)
IMODE SELECTOR 0 I/E Range Mode
IRANGE QUANT 1.37699E-002 Last Measured Current (A)
AREA QUANT 5.00000E+000 Sample Area (cm^2)
EQDELAY QUANT 5.00000E+000 Equil. Time (s)
IRCOMP SELECTOR 0 IRComp
PFCOR QUANT 5.00000E+001 PF Corr. (ohm)
SAMPLINGMODE SELECTOR 1 Sampling Mode
EOC QUANT 0 Open Circuit (V)
SEQUENCER TOGGLE TRUE Run as Sequence
PSTATMODEL IQUANT 64 Pstat Model
PSTATSECTION LABEL IFC1010-20235 Pstat Section
PSTATSERIALNO LABEL 20235 Pstat Serial Number
CTRLMODE IQUANT 1 Control Mode
ELECTROMETER IQUANT 0 RE=0 or CS=1
IESTAB IQUANT 2 I/E Stability

CASPEED	IQUANT	4	Control Amp Speed	
CONVENTION	IQUANT	1	Current Convention	
ICHRANGE	IQUANT	2	Ich Range	
ICHRANGEMODE		TOGGLE	F	Ich Auto Range
ICHOFFSETENABLE		TOGGLE	F	Ich Offset Enable
ICHOFFSET	QUANT	0	Ich Offset (V)	
ICHFILTER	IQUANT	3	Ich Filter	
VCHRANGE	IQUANT	2	Vch Range	
VCHRANGEMODE		TOGGLE	F	Vch Auto Range
VCHOFFSETENABLE		TOGGLE	F	Vch Offset Enable
VCHOFFSET	QUANT	0	Vch Offset (V)	
VCHFILTER	IQUANT	3	Vch Filter	
IERANGELOWERLIMIT		IQUANT	4	I/E Range Lower Limit
IERANGEMODE		TOGGLE	T	I/E AutoRange
IERANGE	IQUANT	8	I/E Range	
ACHSELECT	IQUANT	0	Ach Select	
ACHRANGE	IQUANT	2	Ach Range	
ACHOFFSETENABLE		TOGGLE	F	Ach Offset Enable
ACHOFFSET	QUANT	0	Ach Offset (V)	
ACHFILTER	IQUANT	1	Ach Filter	
SENSECABLEID	IQUANT	12	Sense Cable ID	
PWRCABLEID	IQUANT	12	Power Cable ID	
DCCALDATE	LABEL	1/11/2024	DC Calibration Date	
ACCALDATE	LABEL	2/24/2018	AC Calibration Date	
FRAMEWORKVERSION		QUANT	7.06	Framework Version
INSTRUMENTVERSION		LABEL	4.30	Instrument Version

Gamry Electro Impedance Spectroscopy Header Information

EXPLAIN				
TAG	EISPOT			
TITLE	LABEL	Potentiostatic EIS	Test Identifier	
DATE	LABEL	3/29/2024	Date	
TIME	LABEL	13:35:30	Time	
NOTES	NOTES	1	Notes...	
PSTAT	PSTAT	IFC1010-20235	Potentiostat	
VDC	POTEN	0.00000E+000	T	DC Voltage (V)
FREQINIT		QUANT	2.00000E+004	Initial Freq. (Hz)
FREQFINAL		QUANT	2.00000E-001	Final Freq. (Hz)
PTSPERDEC		QUANT	1.00000E+001	Points/decade
VAC	QUANT	1.00000E+002	AC Voltage (mV rms)	
AREA	QUANT	1.00000E+000	Sample Area (cm^2)	
SPEED	SELECTOR	2	Optimize for:	
ZGUESS		QUANT	1.00000E+002	Estimated Z (ohms)

EOC	QUANT	0	Open Circuit (V)
SEQUENCER	TOGGLE	TRUE	Run as Sequence
PSTATMODEL	IQUANT	64	Pstat Model
PSTATSECTION	LABEL	IFC1010-20235	Pstat Section
PSTATSERIALNO	LABEL	20235	Pstat Serial Number
CTRLMODE	IQUANT	1	Control Mode
ELECTROMETER	IQUANT	0	RE=0 or CS=1
IESTAB	IQUANT	0	I/E Stability
CASPEED	IQUANT	3	Control Amp Speed
CONVENTION	IQUANT	1	Current Convention
ICHRANGE	IQUANT	2	Ich Range
ICHRANGEMODE	TOGGLE	F	Ich Auto Range
ICHOFFSETENABLE	TOGGLE	T	Ich Offset Enable
ICHOFFSET	QUANT	0	Ich Offset (V)
ICHFILTER	IQUANT	4	Ich Filter
VCHRANGE	IQUANT	2	Vch Range
VCHRANGEMODE	TOGGLE	F	Vch Auto Range
VCHOFFSETENABLE	TOGGLE	T	Vch Offset Enable
VCHOFFSET	QUANT	0	Vch Offset (V)
VCHFILTER	IQUANT	4	Vch Filter
IERANGELOWERLIMIT	IQUANT	4	I/E Range Lower Limit
IERANGEMODE	TOGGLE	F	I/E AutoRange
IERANGE	IQUANT	11	I/E Range
ACHSELECT	IQUANT	0	Ach Select
ACHRANGE	IQUANT	2	Ach Range
ACHOFFSETENABLE	TOGGLE	F	Ach Offset Enable
ACHOFFSET	QUANT	0	Ach Offset (V)
ACHFILTER	IQUANT	1	Ach Filter
SENSECABLEID	IQUANT	12	Sense Cable ID
PWRCABLEID	IQUANT	12	Power Cable ID
DCCALDATE	LABEL	1/11/2024	DC Calibration Date
ACCALDATE	LABEL	2/24/2018	AC Calibration Date
FRAMEWORKVERSION	QUANT	7.06	Framework Version
INSTRUMENTVERSION	LABEL	4.30	Instrument Version

Gamry Pulse Electroplating Header Information

EXPLAIN			
TAG	REPEATING_CHRONOA		
TITLE	LABEL	Chronoamperometry Scan	Test Identifier
DATE	LABEL	3/29/2024	Date
TIME	LABEL	13:59:59	Time
NOTES	NOTES	1	Notes...
PSTAT	PSTAT	IFC1010-20235	Potentiostat

VSTEP1	POTEN	-1.97000E-001	F	Step 1 Voltage (V)
TSTEP1	QUANT	1.00000E+000		Step 1 Time (s)
VSTEP2	POTEN	-2.00000E+000	F	Step 2 Voltage (V)
TSTEP2	QUANT	1.00000E+001		Step 2 Time (s)
SAMPLETIME	QUANT	2.50000E-003		Sample Period (s)
DECIMATE	TOGGLE	F		Decimate
ILIMIT	QUANT	5.00000E+003		Limit I (mA/cm^2)
AREA	QUANT	1.00000E+001		Sample Area (cm^2)
IMODE	SELECTOR	0		I/E Range Mode
IRANGE	QUANT	1.00000E+003		Max Current (mA)
EQDELAY	QUANT	0.00000E+000		Equil. Time (s)
IRCOMP	SELECTOR	0		IRComp
PFCOR	QUANT	5.00000E+001		PF Corr. (ohm)
CYCLES	IQUANT	2		Cycles (#)
SAMPLINGMODE	SELECTOR	1		Sampling Mode
ELECTRODETYPE	SELECTOR	0		Electrode Type
STRIPPING	TOGGLE	F		Used for Stripping
POLARITY	TOGGLE	F		Signal Polarity
LINEFREQ	IQUANT	60		Line Frequency (Hz)
INTPERIOD	QUANT	NIL		Integration Period (s)
SEQUENCER	TOGGLE	TRUE		Run as Sequence
PSTATMODEL	IQUANT	64		Pstat Model
PSTATSECTION	LABEL	IFC1010-20235		Pstat Section
PSTATSERIALNO	LABEL	20235		Pstat Serial Number
CTRLMODE	IQUANT	1		Control Mode
ELECTROMETER	IQUANT	0		RE=0 or CS=1
IESTAB	IQUANT	0		I/E Stability
CASPEED	IQUANT	4		Control Amp Speed
CONVENTION	IQUANT	1		Current Convention
ICHRANGE	IQUANT	2		Ich Range
ICHRANGEMODE	TOGGLE	T		Ich Auto Range
ICHOFFSETENABLE	TOGGLE	F		Ich Offset Enable
ICHOFFSET	QUANT	0		Ich Offset (V)
ICHFILTER	IQUANT	3		Ich Filter
VCHRANGE	IQUANT	3		Vch Range
VCHRANGEMODE	TOGGLE	T		Vch Auto Range
VCHOFFSETENABLE	TOGGLE	F		Vch Offset Enable
VCHOFFSET	QUANT	0		Vch Offset (V)
VCHFILTER	IQUANT	3		Vch Filter
IERANGELOWERLIMIT	IQUANT	4		I/E Range Lower Limit
IERANGEMODE	TOGGLE	T		I/E AutoRange
IERANGE	IQUANT	12		I/E Range
ACHSELECT	IQUANT	0		Ach Select
ACHRANGE	IQUANT	2		Ach Range

ACHOFFSETENABLE	TOGGLE	F	Ach Offset Enable
ACHOFFSET	QUANT	0	Ach Offset (V)
ACHFILTER	IQUANT	1	Ach Filter
SENSECABLEIDIQUANT	12	Sense Cable ID
PWRCABLEID	IQUANT	12	Power Cable ID
DCCALDATE	LABEL	1/11/2024	DC Calibration Date
ACCALDATE	LABEL	2/24/2018	AC Calibration Date
FRAMEWORKVERSION	QUANT	7.06	Framework Version
INSTRUMENTVERSION	LABEL	4.30	Instrument Version

Gamry Square Wave Header Information

EXPLAIN
TAG	SQUARE_WAVE
TITLE	LABEL	Square Wave Voltammetry	Test Identifier
DATE	LABEL	3/29/2024	Date
TIME	LABEL	13:54:26	Time
NOTES NOTES 1	Notes...

PSTAT	PSTAT	IFC1010-20235	Potentiostat
VINIT	QUANT	-1.97000E-001	Initial E (V)
VFINAL	QUANT	-2.75000E+000	Final E (V)
STEPSIZE	QUANT	2.00000E+000	Step Size (mV)
TIMERRES	QUANT	1.00000E-004	&Timer Resolution
FREQUENCY	QUANT	1.00000E+001	Frequency (Hz)
PULSESIZE	QUANT	1.00000E+002	Pulse Size E (mV)
PULSEON	QUANT	1.00000E-001	Pulse &Time (s)
NOISEREJECT	TOGGLE	T	Noise Rejection
AREA	QUANT	1.00000E+000	Sample Area (cm^2)
IMODE	SELECTOR	1	I/E Range Mode
IRANGE	QUANT	1.00000E+003	Max Current (mA)
EQDELAY	IQUANT	5	Equil. Time (s)
IRCOMP	SELECTOR	0	IRComp
PFCOR	QUANT	5.00000E+001	PF Corr. (ohm)
CYCLES	IQUANT	1278	C&ycles (#)
ELECTRODETYPE	SELECTOR	0	Electrode Type
STRIPPING	TOGGLE	F	Used for Stripping
POLARITY	TOGGLE	F	Signal Polarity
LINEFREQ	IQUANT	60	Line Frequency (Hz)
INTPERIOD	QUANT	0.0166667	Integration Period (s)
SEQUENCER	TOGGLE	TRUE	Run as Sequence
PSTATMODEL	IQUANT	64	Pstat Model
PSTATSECTION	LABEL	IFC1010-20235	Pstat Section
PSTATSERIALNO	LABEL	20235	Pstat Serial Number
CTRLMODE	IQUANT	1	Control Mode

ELECTROMETER	IQUANT	0	RE=0 or CS=1
IESTAB	IQUANT	2	I/E Stability
CASPEED	IQUANT	6	Control Amp Speed
CONVENTION	IQUANT	0	Current Convention
ICHRANGE	IQUANT	2	Ich Range
ICHRANGEMODE	TOGGLE	F	Ich Auto Range
ICHOFFSETENABLE	TOGGLE	F	Ich Offset Enable
ICHOFFSET	QUANT	0	Ich Offset (V)
ICHFILTER	IQUANT	3	Ich Filter
VCHRANGE	IQUANT	2	Vch Range
VCHRANGEMODE	TOGGLE	F	Vch Auto Range
VCHOFFSETENABLE	TOGGLE	F	Vch Offset Enable
VCHOFFSET	QUANT	0	Vch Offset (V)
VCHFILTER	IQUANT	3	Vch Filter
IERANGELOWERLIMIT	IQUANT	4	I/E Range Lower Limit
IERANGEMODE	TOGGLE	F	I/E AutoRange
IERANGE	IQUANT	12	I/E Range
ACHSELECT	IQUANT	0	Ach Select
ACHRANGE	IQUANT	2	Ach Range
ACHOFFSETENABLE	TOGGLE	F	Ach Offset Enable
ACHOFFSET	QUANT	0	Ach Offset (V)
ACHFILTER	IQUANT	1	Ach Filter
SENSECABLEID	IQUANT	12	Sense Cable ID
PWRCABLEID	IQUANT	12	Power Cable ID
DCCALDATE	LABEL	1/11/2024	DC Calibration Date
ACCALDATE	LABEL	2/24/2018	AC Calibration Date
FRAMEWORKVERSION	QUANT	7.06	Framework Version
INSTRUMENTVERSION	LABEL	4.30	Instrument Version

Python Script for Automated Cyclic Voltammetry Batch Analysis

```
# -*- coding: utf-8 -*-
"""
Created on Tue Jan 21 19:18:46 2025

@author: Diana Horangic
"""
import os
import matplotlib.pyplot as plt
import numpy as np
from AUX_FUNCTIONS import *

Home = ".\DEMONSTRATION_DATASET"
Analytic_Integrals = True #Analytical integration
Data_Interval = (2.75, 0.3) #Voltage
Operator_Color = {"Chris" : 'red', "Steven" : 'green', "Kat" : 'blue'}
```

```
#There is also code written to run through a large file directory of data, and output it to large excel documents or CSV files.
#It was written to output the analysis in columns labeled:
    #["TEMPERATURE", "DATE", "SCAN SPEED", "CURVE NUMBER", "V1 POINT", "V2 POINTS", "HALWAY DIP POINT", "AREA"]
#Examples are available with this demo, I have set aside some of those excel spreadsheets.

if __name__ == "__main__":
    #1. Choose a compound - "TiCl", "ZrCl", "TiF" (technically tetra!)
    Compound = "TiCl"
    #2. Choose a range of CV scan rates (mV/sec) to look at, from 25 to 5k
    CV_Rates = ["25", "100", "500"]
    #3. Load the data from different operators.
    Operators = os.listdir(os.path.join(Home, Compound))
    print(f"The different people who took data with this compound are: {Operators}")
    #4. Either look at each scan, or run through the data taken for different operators.
    for op in Operators:
        file_directory = os.path.join(Home, Compound, op)
        file_locations = [os.path.join(file_directory, f"CV{CV}.txt") for CV in CV_Rates]
        for idx, file_ in enumerate(file_locations):
            CV = CV_Rates[idx]
            assert os.path.isfile(file_), f"The file path is {file_}"
            try:
                V, C, T = read_files(file_)
            except Exception as e:
                print(f"For file {file_}, error! {e}")
            V_s, C_s, T_s, Stored_Mins = process_curves(V, C, T, Data_Interval, file_)
            for curve_num in range(1, len(V_s)):
                minima, X, Y, list_, tolerance = condensed_analysis(V, C, V_s, C_s, curve_num, Stored_Mins, CV, file_)

                plt.figure(figsize = (10,8))
                for tup_ in minima:
                    plt.axvline(x = X[tup_[0]], color='deeppink', linestyle = "--")
                    plt.axvline(x = X[tup_[1]], color='deeppink', linestyle = "--")
                plt.plot(X, Y, color=Operator_Color[op], label = f"Number of dips found by preliminary analysis: {len(tup_)}", linewidth = 3.0)
                plt.xlabel("Voltage (V)")
                plt.ylabel("Current (A)")
                plt.title(f"Prelim. Analysis: Curve #{curve_num}, at scan speed {CV} mV/sec, performed by operator {op} with compound {Compound}")
                plt.legend()
                plt.show()
```

```
        if len(list_) != 0:
            plt.figure(figsize = (10,8))
            range_ = 15
            plt.plot(X, Y, color=Operator_Color[op], label = f"Number of dips found by
refined analysis: {len(list_)}", linewidth = 3.0)
            for tup_ in list_:
                idx_triplet, m, b, ab, cur, half1, half2, area = tup_
                start, finish = idx_triplet[0], idx_triplet[1] + range_
                C_fit = (X[start : finish] * m) + b
                C_original = Y[start : finish]
                plt.axvline(x = ab[0], color='deeppink', linestyle = "--")
                plt.axvline(x = ab[1], color='deeppink', linestyle = "--")
                plt.plot(X[start : finish], C_fit, color = 'black', linestyle = ':')
                between_ = int ( (float( (start) + (finish)) / 2.0)  )
                xmax = X[between_]
                ymax = Y[between_]
                x2 = X[between_] #- 0.15 * np.max(X[start:finish])
                y2 = Y[between_] - 0.15 * np.max(C_original)
                C_fit_half = C_fit[int( float(len(C_fit)) / 2.0)]
                C_org_half = C_original[int( float(len(C_fit)) / 2.0)]
                bool_valid_area = True
                if C_org_half > C_fit_half or abs(C_org_half - C_fit_half) <= tolerance:
                    bool_valid_area = False
                sci_area = f"{area:.4e}"
                plt.annotate(f'{bool_valid_area}', xy=(xmax, ymax), xytext=(xmax,
ymax),
                             bbox=dict(boxstyle='square', fc='white', ec='black'),
                             arrowprops=dict(facecolor='black', arrowstyle='-'))
                plt.annotate(f'{sci_area}', xy=(x2, y2), xytext=(x2, y2),
                             bbox=dict(boxstyle='square', fc='white', ec='black'),
                             arrowprops=dict(facecolor='black', arrowstyle='-'))
            plt.xlabel("Voltage (V)")
            plt.ylabel("Current (A)")
            plt.title(f"Refined Analysis: Curve #{curve_num}, at scan speed {CV}
mV/sec, performed by operator {op} with compound {Compound}")
            plt.legend()
            plt.show()
```
